# Exogenous Melatonin in the Culture Medium Does Not Affect the Development of In Vivo-Derived Pig Embryos but Substantially Improves the Quality of In Vitro-Produced Embryos

**DOI:** 10.3390/antiox11061177

**Published:** 2022-06-15

**Authors:** Cristina A. Martinez, Cristina Cuello, Inmaculada Parrilla, Carolina Maside, Guillermo Ramis, Josep M. Cambra, Juan M. Vazquez, Heriberto Rodriguez-Martinez, Maria A. Gil, Emilio A. Martinez

**Affiliations:** 1 Department of Medicine and Animal Surgery, Faculty of Veterinary Medicine, International Excellence Campus for Higher Education and Research (CMN), University of Murcia, 30100 Murcia, Spain; parrilla@um.es (I.P.); carolina.maside@um.es (C.M.); josepmiquel.cambra@um.es (J.M.C.); vazquez@um.es (J.M.V.); mariagil@um.es (M.A.G.); emilio@um.es (E.A.M.); 2 Department of Biomedical & Clinical Sciences (BKV), BKH/Obstetrics & Gynaecology, Faculty of Medicine and Health Sciences, Linköping University, SE-58185 Linköping, Sweden; heriberto.rodriguez-martinez@liu.se; 3 Institute for Biomedical Research of Murcia (IMIB-Arrixaca), 30120 Murcia, Spain; guiramis@um.es; 4 Department of Biology, Faculty of Sciences, University of Girona, 17003 Girona, Spain; 5 Department of Animal Production, Faculty of Veterinary Medicine, International Excellence Campus for Higher Education and Research (CMN), University of Murcia, 30100 Murcia, Spain

**Keywords:** melatonin, in vitro fertilization, reactive oxygen species, in vivo-derived embryos, CRISPR/Cas9, apoptosis, inner cell mass, pig

## Abstract

Cloned and transgenic pigs are relevant human disease models and serve as potential donors for regenerative medicine and xenotransplantation. These technologies demand oocytes and embryos of good quality. However, the current protocols for in vitro production (IVP) of pig embryos give reduced blastocyst efficiency and embryo quality compared to in vivo controls. This is likely due to culture conditions jeopardizing embryonic homeostasis including the effect of reactive oxygen species (ROS) influence. In this study, the antioxidant melatonin (1 nM) in the maturation medium, fertilization medium, or both media was ineffective in enhancing fertilization or embryonic development parameters of in vitro fertilized oocytes. Supplementation of melatonin in the fertilization medium also had no effect on sperm function. In contrast, the addition of melatonin to the embryo culture medium accelerated the timing of embryonic development and increased the percentages of cleaved embryos and presumed zygotes that developed to the blastocyst stage. Furthermore, it increased the number of inner mass cells and the inner mass cell/total cell number ratio per blastocyst while increasing intracellular glutathione and reducing ROS and DNA damage levels in embryos. Contrarily, the addition of melatonin to the embryo culture medium had no evident effect on in vivo-derived embryos, including the developmental capacity and the quality of in vivo-derived 4-cell embryos or the percentage of genome-edited in vivo-derived zygotes achieving the blastocyst stage. In conclusion, exogenous melatonin in the embryo culture medium enhances the development and quality of in vitro-derived embryos but not in in vivo-derived embryos. Exogenous melatonin is thus recommended during embryo culture of oocytes matured and fertilized in vitro for improving porcine IVP efficiency.

## 1. Introduction

Efficient procedures for the in vitro production (IVP) of embryos are very important for basic research and biomedical applications. Many studies have aimed to improve IVP systems and increase the production of good-quality embryos [1,2,3]. However, IVP efficiency remains suboptimal, with monospermic fertilization and blastocyst formation rates of approximately 45% and 35%, respectively [4], in conspicuous contrast to in vivo-derived embryos. There is, obviously, a deficient culture environment for in vitro maturation (IVM), in vitro fertilization (IVF), and/or in vitro culture (IVC) [1,5,6,7]. One of the main concerns during IVP is greater exposure to light [8], oxygen [9], and oxidant compounds produced by the metabolic processes of gametes and embryos [10], all inducing oxidative stress in the gametes and embryos, via increases in reactive oxygen species (ROS) levels in the culture media. This fact can lead to the degradation of biological molecules and, consequently, to the oocyte and/or embryo death [10,11,12]. Certainly, countermeasures have been assayed, particularly the addition of antioxidant compounds to the culture media [13,14,15,16,17,18], albeit with meager success.

The hormone melatonin (*N*-acetyl-5-methoxytryptamine) has been shown to down-regulate and upregulate the expression of proapoptotic genes and antiapoptotic and antioxidant genes, respectively, which is accompanied by a reduction in ROS production. Melatonin has been extensively used to protect biological molecules from oxidative damage in humans and different animal species, and numerous murine and bovine studies using in vitro matured and fertilized oocytes have reported that the addition of melatonin to IVM and IVC media positively affects oocyte maturation and embryonic development and quality [19]. In pigs, melatonin has also been added to IVM and/or IVC media to enhance embryo IVP, mostly using embryos obtained via parthenogenetic activation or somatic cell nuclear transfer (SCNT), with positive effects on IVP outcomes although the results are not completely consistent [20,21,22,23,24,25,26,27,28]. In contrast, a very limited number of studies have determined the effect of melatonin using oocytes matured and fertilized in vitro and with controversial results [29,30,31]. Moreover, the effects of sequential supplementation of IVM, IVF, and IVC media with melatonin on maturation, fertilization, and embryonic development parameters and the timing of embryonic development have not yet been established. Additionally, the impact of melatonin during embryo culture on the development and kinetics of in vivo-derived embryos remains unknown.

On the other hand, zygote genome-editing (ZGE) with nucleases is an important technology used in animal production and biomedicine. Currently, most studies use in vivo-derived zygotes because zygote quality is a key factor contributing to the success of this technology [32]. ZGE might imply an increase in embryo vulnerability to the culture environment compared to nonmanipulated embryos, as porcine embryos produced by SCNT are more sensitive to culture conditions and the environment than those produced by IVF [33]. In this scenario, melatonin might decrease the environmental impact of stressors on culture media, increasing the competence of edited in vivo-derived zygotes to reach viable blastocysts after in vitro culture. This property would also be crucial for the use of other emerging technologies, such as the interspecies blastocyst complementation approach following ZGE [34,35,36].

The objectives of this study were (1) to evaluate the effect of sequential addition of melatonin to IVP media on porcine maturation, fertilization, and embryo production outcomes and (2) to determine the effect of melatonin supplementation of IVC medium on the developmental timing of in vitro-produced porcine embryos. Moreover, we intended to determine its effect when added during IVC on (3) the developmental capacity and kinetics of in vivo-derived 4-cell porcine embryos and on the quality of the resulting blastocysts, and (4) the developmental competence of Clustered Regularly Interspaced Short Palindromic Repeats/CRISPR-associated protein 9 (CRISPR/Cas9)-injected in vivo-derived porcine zygotes.

## 2. Materials and Methods

### 2.1. Ethics

The experiments were reviewed and approved by the Ethical Committee of the University of Murcia, Spain (ID: 69/2014) and by the Autonomous Community of the Region of Murcia, Spain (ID: A1320141201) according to European Parliament legislation (2010/63/EU directive).

### 2.2. Chemicals

All chemicals used were purchased from the Sigma-Aldrich Chemical Company (Madrid, Spain) unless specified otherwise.

### 2.3. In Vitro Embryo Production

#### 2.3.1. Collection of Cumulus-Oocyte Complexes

Prepubertal crossbred gilts slaughtered at a commercial slaughterhouse (El Pozo S.A., Murcia, Spain) were used as oocyte sources. Immediately after collection, the ovaries were shipped at 35 °C to our laboratory in physiological serum containing kanamycin (70 µg/mL) for immediate cumulus-oocyte complex (COC) obtention. The COCs were collected in Tyrode’s medium containing lactate, *N*-2-hydroxyethyl piperazine ethanesulfonic acid (HEPES), and polyvinyl alcohol (TL-HEPES-PVA) as previously reported [37,38] at 38 °C by sectioning the surface of antral follicles with diameters ranging from 3–6 mm using a disposable sterile blade.

#### 2.3.2. In Vitro Maturation

Oocytes with several layers of cumulus cells and with uniform black cytoplasm were matured in an IVM medium (TCM-199; Gibco Life Technologies S.A., Barcelona, Spain) containing cysteine (0.57 mM), PVA (0.1%), epidermal growth factor (10 ng/mL), penicillin G potassium (75 µg/mL), and streptomycin sulfate (50 µg/mL)]. The COCs (75–80 per dish) were incubated for 20–22 h in 500 µL of IVM medium containing 10 IU/mL equine chorionic gonadotropin (eCG; Folligon, Intervet International B.V., Boxxmeer, The Netherlands) and 10 IU/mL human chorionic gonadotropin (hCG; Veterin Corion, Divasa Farmavic S.A., Barcelona, Spain). Then, the COCs were cultured for an additional 20–22 h in a fresh IVM medium in the absence of hormones. Maturation was performed under paraffin oil [39] at 38.5 °C with 5% CO_2_ and saturated humidity.

#### 2.3.3. Sperm Freezing and Thawing

Semen from a boar was frozen and thawed as previously reported [40]. Briefly, the sperm-rich fraction was extended (1:1) in Beltsville thawing solution (BTS) [41] and incubated at 17 °C for 3 h before centrifugation (2400× *g*; 3 min). Then, the supernatants were discarded, and the pellets were extended to 1.5 × 10^9^ sperm/mL in lactose egg yolk (LEY) medium and cooled at 5 °C for 2 h. Subsequently, the spermatozoa were extended to 1 × 10^9^ sperm/mL in LEY-Glycerol-Orvus Es Paste medium and packed into 0.5 mL straws before freezing in a biofreezer and immersion in liquid nitrogen (LN_2_). Straws were thawed in a water bath at 37 °C for 20 s.

#### 2.3.4. In Vitro Fertilization

Before coincubation with spermatozoa, matured oocytes were denuded and washed with IVM and IVF media. The IVF medium consisted of Tris-buffered solution [42] supplemented with caffeine (2 mM) and bovine serum albumin (BSA; 0.2 mg/mL). The oocyte-sperm coincubation was performed as previously reported [43]. Briefly, groups of 30 denuded oocytes were incubated in 50 µL drops of IVF medium under paraffin oil at 38.5 °C with 5% CO_2_ in the air and maximum humidity until sperm addition (approximately 30 min). Frozen spermatozoa from two straws were thawed and pooled in each replicate and washed three times (1900× *g* for 3 min) in phosphate-buffered saline (PBS; Gibco, Grand Island, NY, USA) containing BSA (4 mg/mL). Then, the pellet was extended in IVF medium, and 50 µL of extended spermatozoa were added to the 50 µL drop containing the oocytes. The sperm concentration used was 30 × 10^4^ spermatozoa/mL. The oocyte-spermatozoa incubation was performed for 5 h under paraffin oil at 38.5 °C with 5% CO_2_ and maximum humidity.

#### 2.3.5. Culture of Embryos

At the end of oocyte-sperm coincubation, the presumed zygotes were repeatedly pipetted to eliminate spermatozoa attached to the zona pellucida (ZP) in IVC medium (North Carolina State University-23; NCSU-23) [44] containing BSA (0.4%). Then, groups of 40 presumptive zygotes were cultured in 500 µL of IVC medium containing pyruvate (0.3 mM) and lactate (4.5 mM) for 2 days and then in 500 µL of fresh IVC medium supplemented with glucose (5.5 mM) for an additional 5 days. The culture was performed under paraffin oil at 38.5 °C in 5% CO_2_ in air and maximum humidity.

#### 2.3.6. Assessment of IVM, IVF, and IVC Parameters

The nuclear maturation status and the fertilization outcomes were assessed by phase-contrast microscopy after 44 h of maturation and 18 h post-fertilization, respectively, after previous oocyte fixation with acetic acid and staining with lacmoid [38]. Oocytes with nucleoli and chromatin surrounded by an evident nuclear membrane and those with chromosomes in metaphase (M) without an observable polar body (PB) were classified as germinal vesicle and MI oocytes, respectively. Oocytes with a clearly identifiable M-plate and an extruded PB were considered mature (MII). The maturation rate was the ratio of MII oocytes to the overall number of examined oocytes. Presumed zygotes with one or more male pronuclei and two PBs were considered penetrated. The following IVF parameters were evaluated: penetration rate (percentage of mature oocytes penetrated), monospermic rate (percentage of mature monospermic oocytes among total penetrated oocytes), and IVF efficiency (percentage of monospermic oocytes among total mature oocytes inseminated). Oocytes with an anomalous cytoplasmic morphology were classified as degenerated and eliminated from the experiments.

In vitro embryonic development was assessed using stereomicroscopy during culture. Embryos that reached the 2- to 4-cell stage on day 2 of culture were considered cleaved, and embryos with a clear blastocoel on days 5–7 of culture were defined as blastocysts. The cleavage rate was the percentage of inseminated oocytes that developed to the 2- to 4-cell stage. The blastocyst formation rate was the proportion of cleaved embryos that progressed to blastocysts. The total IVC efficiency was the proportion of the presumed zygotes that developed to the blastocyst stage.

The total cell number (TCN) per blastocyst was used as an embryo quality marker. The blastocysts were fixed in paraformaldehyde, washed with PBS-BSA, and transferred into microdrops of Vectashield (Vector, Burlingame, CA, USA) supplemented with Hoechst 33,342 [38]. The TCN per blastocyst was the number of blue nuclei observed under a fluorescence microscope (excitation filter: 330 to 380 nm).

### 2.4. Evaluation of Sperm Status

#### 2.4.1. Sperm Viability (Integrity of the Plasma Membrane and Acrosome)

For determination of sperm viability, sperm samples (90 μL) containing 30 × 10^4^ sperm/mL were incubated (in the dark) at 38 °C for 10 min with Hoechst 33342, propidium iodide (PI), and fluorescein-conjugated peanut agglutinin (PNA-FITC) as previously described [45]. The samples were then extended in PBS and 10,000 spermatozoa were examined by flow cytometry. Viable spermatozoa were considered positive for Hoechst and negative for both PI and PNA-FITC.

#### 2.4.2. Plasma Membrane Destabilization

Plasma membrane fluidity was determined as previously described [45]. Briefly, 90 μL samples containing 30 × 10^4^ spermatozoa/mL were extended to 1 mL with PBS supplemented with Hoechst 33,342 and Yo-Pro-1 (YOPRO; Molecular Probes Europe BV, Leiden, The Netherlands) and incubated (in the dark) at 38 °C for 8 min. Then, the samples were incubated with merocyanine 540 (MERO; Molecular Probes Europe BV) for another 2 min and analyzed by flow cytometry. A total of 10,000 spermatozoa were counted. Cells positive for Hoechst and MERO and negative for YOPRO were considered viable spermatozoa with increased fluidity of the plasma membrane.

#### 2.4.3. Apoptosis

A 90 µL suspension containing 30 × 10^4^ spermatozoa/mL was incubated at 22 °C (in the dark) for 15 min with Annexin-V binding buffer, Hoechst 33342, PI, and Annexin V-FITC (AV; Life Technologies-Molecular Probes, TermoFisher Scientific, Waltham, MA, USA) as previously described [46]. Then, the samples were mixed with Annexin-V binding buffer and analyzed by flow cytometry. A total of 10,000 spermatozoa were counted, and those spermatozoa negative for PI and positive for AV staining were considered viable with early apoptotic-like changes.

### 2.5. In Vivo Derived Embryos

#### 2.5.1. Animals

Crossbred sows (2–6 parity) with lactation periods of 21 to 24 days were used for the in vivo studies. The sows were weaned and located in individual cages in a programmed, ventilated pregnancy room at a commercial farm (Agropor SA, Murcia, Spain). Semen was manually collected from boars located at a commercial artificial insemination center. All animals were provided water ad libitum and were fed a diet conforming to their nutritional needs.

#### 2.5.2. Superovulation Treatment, Estrous Detection, and Insemination

Donor sows were superovulated with exogenous gonadotropins as previously described [47]. Briefly, eCG was intramuscularly administered (1000 IU) 24 h after weaning, and hCG (750 IU; im) was administered at 72–96 h after eCG injection at the onset of estrus. Estrous detection started at 24 h post-weaning. Sows with an immobilization reflex in the presence of a boar were noted to be in estrus. Sows were inseminated (post-cervical insemination) at 6 and 24 h after the onset of estrus with 1.5 × 10^9^ fresh sperm extended in 50 mL of BTS.

#### 2.5.3. Collection and Evaluation of Embryos

Embryos were obtained by laparotomy at days 2 or 3 (day 0 = onset of estrus) to collect zygotes or four-cell embryos, respectively. Sedation and general anesthesia were induced with azaperone (2 mg/kg, i.m.; Stresnil^®^, Sanochemia Pharmazeutika A.G., Landegger Strasse, Austria) and sodium thiopental (7 mg/kg i.v.; B. Braun VetCare SA, Barcelona, Spain), respectively. Isoflurane (3–5% in air; IsoFlo^®^, Zoetis Spain S.L., Madrid, Spain) was used to maintain general anesthesia. Embryos were retrieved into 50 mL sterile tubes by washing the oviducts from the utero-tubal junction toward the infundibulum with TL-HEPES-PVA (30 mL; 38.5 °C). The recovered structures were washed, transferred into sterilized Eppendorf tubes, and immediately shipped in TL-HEPES-PVA at 38.5 °C to our laboratory. The recovered structures were then evaluated by stereomicroscopy to assess their developmental stage and quality. Structures with a single cell without sperm attached to the ZP and only one visible PB were considered oocytes and excluded from the experiment. Structures collected on day 2 with a single cell and two clearly visible PBs or those recovered on day 3 with 3–4 similarly sized blastomeres and no fragments were considered zygotes or 4-cell embryos, respectively.

#### 2.5.4. Cytoplasmic Microinjection of Zygotes

The previously reported laser-assisted cytoplasmic microinjection procedure was utilized for zygote injection [48]. Briefly, zygotes were individually attached to the holding pipette and evaluated for quality. Only zygotes with two PBs and good morphology were injected. The zygotes were repositioned with the PBs oriented at 6 or 12 o’clock and a perforation was then created in the ZP at 3 o’clock with the laser. The injection pipette was introduced through the hole and positioned into the zygote while applying negative pressure until a cytoplasmic portion was suctioned into the injection pipette as a sign of rupture of the plasmalemma. Then, the suction-retrieved cytoplasm and the solution containing Cas9 mRNA (100 ng/µL) and sgRNA (20 ng/µL) were injected into the cytoplasm by applying positive pressure to the injection pipette.

#### 2.5.5. Culture of In Vivo-Derived Embryos and Assessment of Embryonic Development

Groups of 20 in vivo-derived embryos (zygotes or 4-cell embryos) were cultured in 500 μL of NCSU-23 medium under the same culture conditions specified above for the IVP procedure. The embryonic developmental stage was evaluated at 2 and 7 days (injected zygotes) or at 3 and 4 days (4-cell embryos) of in vitro culture. The cleavage rate was the percentage of zygotes that developed to 2- to 4-cell stage embryos. The blastocyst formation rate was the percentage of cleaved embryos that progressed to blastocysts. The total efficiency was the proportion of cultured zygotes or 4-cell embryos that progressed to the blastocyst stage during culture.

### 2.6. Vitrification and Warming

Vitrification was conducted as previously reported [49]. Before vitrification, groups of 5 to 7 blastocysts were equilibrated in TL-HEPES-PVA containing 7.5% dimethyl sulfoxide (DMSO) and 7.5% ethylene glycol (EG) for 3 min and in TL-HEPES-PVA containing 16% DMSO, 16% EG and 0.4 M sucrose for 1 min. After loading the embryos in super open pulled straws (SOPS; Minitüb, Tiefenbach, Germany), the straws were immediately placed in LN_2_. Embryos were warmed using the direct warming method [49]. Briefly, the vitrified embryos were transferred to TL-HEPES-PVA containing 0.13 M sucrose at 38.5 °C for 5 min. Vitrified-warmed embryos were cultured for 24 h in an IVC medium containing fetal calf serum (10%) under the same culture conditions indicated above. Viable blastocysts were considered those blastocysts with good morphology that re-expanded at the end of the post-warming culture. The survival and hatching rates were the percentage of viable blastocysts and the percentage of embryos that developed to the hatching-hatched blastocyst stage at the end of the post-warming culture, respectively.

### 2.7. Differential Immunostaining of Blastocysts

The simultaneous staining of cells of the trophectoderm (TE) and inner cell mass (ICM) was performed using indirect immunofluorescence [50]. Briefly, in vitro-produced blastocysts were fixed as described above for the determination of TCN, permeabilized with Triton X-100 and Tween-20, washed (PBS-BSA), and denatured with HCl (2 N) for 20 min and Tris-HCl (pH 8.5) for 10 min. Then, the blastocysts were washed again (PBS–BSA) and incubated in PBS supplemented with normal donkey serum and Tween-20 (blocking solution), for 5 h. After washing (PBS–BSA), the embryos were incubated in the primary CDX2 antibody (1:200 dilution in antibody diluent; Biogenex, San Ramon, CA, USA) for 36 h. The embryos were then washed (PBS–BSA) and incubated in a blocking solution containing a diluted (1:1000) donkey anti-mouse IgG-Alexa Fluor 568 antibody (Invitrogen, ThermoFisher Scientific, Waltham, MA, USA) for 30 min. After two wash steps of 15 min in PBS-BSA, the embryos were placed in Vectashield (Vector Labs, Burlingame, CA, USA) containing Hoechst 33342. Embryos were evaluated by fluorescence microscopy to determine the number of TE cells (red nuclei) and TCNs (blue nuclei) using 536 nm and 330–380 nm excitation filters, respectively. In merged images, purple and blue colors were designated TE and ICM cells, respectively. The ratio of ICM cells per blastocyst was defined as the number of ICM cells to the total number of cells.

### 2.8. Determination of Intracellular Levels of Glutathione (GSH) and Reactive Oxygen Species (ROS)

The levels of GSH and ROS were assessed in cleaved IVP embryos using 4-chloromethyl- 6,8-difluoro-7-hydroxycoumarin (CellTracker Blue; CMF2HC; Invitrogen) and 2′,7′-dichlorodihydrofluorescein diacetate (CM-H2DCFDA; Invitrogen), respectively [18]. The embryos were washed and then incubated at 38.5 °C for 30 min in TL-HEPES-PVA containing H2DCFDA and CellTracker Blue. Embryos were then washed again, transferred into TL-HEPES-PVA medium, and directly evaluated under a fluorescence microscope with excitation filters of 460 nm and 370 nm for ROS and GSH measurements, respectively. The fluorescence intensity in each embryo was assessed using ImageJ software (Version 1.51h; National Institutes of Health, Bethesda, MD, USA).

### 2.9. Evaluation of DNA Damage in Blastocysts

DNA damage was assessed in blastocysts with the APO-BrdUTM terminal deoxynucleotidyl transferase dUTP nick end labeling (TUNEL) Assay Kit (Invitrogen) as described in previous studies [51]. Briefly, after fixation in paraformaldehyde, the embryos were permeabilized with Triton X-100 and Tween 20. Positive control embryos were incubated in DNase I. Blastocysts were placed in PBS-BSA-Tween 20 medium and incubated in TUNEL reaction medium and then with mouse anti-BrdU-Alexa Fluor 488 antibody. Thereafter, the embryos were washed, transferred to microdroplets of Vectashield-Hoechst 33,342 solution, and evaluated under a fluorescence microscope using excitation filters of 465–495 nm and 330–380 nm for green and blue fluorescence, respectively. Cells with green nuclei were considered TUNEL+ cells. The DNA damage rate was the percentage of TUNEL+ cells to the total Hoechst-stained nuclei (blue fluorescence).

### 2.10. Melatonin Preparation

Stock solutions with 100 nM melatonin (Sigma-Aldrich Cat# M5250, Madrid, Spain) in every medium used in the experiments (i.e., on IVM, IVF, and IVC medium) containing 0.001% ethanol were frozen at −20 °C until use. Then, each stock solution was added to IVM, IVF, and/or IVC media to reach a final concentration of 1 nM melatonin. The concentration of 1 nM melatonin was used in all experiments because it was reported to be the optimal concentration in previous studies [22,23,27,29,30,52].

### 2.11. Experimental Design

#### 2.11.1. Experiment 1. Effects of Melatonin Supplementation of IVP Media on Fertilization and Embryonic Development Parameters

This experiment investigated the effect of melatonin on the in vitro maturation, fertilization, and culture of oocytes or embryos. Mature oocytes were fertilized and cultured for 18 h or 7 days to determine the fertilization and embryonic development outcomes, respectively. First, we evaluated the effects of melatonin added to the IVM and IVF media in all possible combinations (IVM + IVF+, IVM+, and IVF+ groups) on IVP outcomes. Oocytes matured and fertilized in the absence of melatonin constituted the controls. We also investigated the effect of melatonin added to the IVF medium during gamete coincubation on the functionality of spermatozoa. Gamete coincubation was performed under the same conditions described above. Sperm samples were collected at 10, 60, and 180 min of coincubation, after removing the oocytes, to assess sperm viability, plasma membrane destabilization, and apoptosis. Second, melatonin was either added to or absent from (controls) both IVM and IVC media (IVM + IVC+ group) or only to IVC medium (IVC+ group) to determine its effect during embryo culture on embryonic development parameters. A total of 3056 oocytes in six replicates were used in these experiments.

#### 2.11.2. Experiment 2. Effects of Melatonin during IVM and IVC on the Quality of Blastocysts Produced In Vitro: Number of Trophectoderm (TE) and Inner Cell Mass (ICM) Cells and ICM/TCN Ratio

Oocytes were matured in IVM medium in the presence or absence of melatonin and inseminated with thawed sperm. Then, presumed zygotes were cultured in IVC medium containing melatonin (IVM + IVC+, and IVC+ groups). On day 7 of culture, the resulting blastocysts were subjected to differential immunostaining to determine the TCN, the number of TE and ICM cells, and the ICM/TCN ratio as parameters of embryo quality. Blastocysts produced in vitro in the absence of melatonin were used as controls. Forty-five blastocysts were used in three replicates.

#### 2.11.3. Experiment 3. Effects of Melatonin during IVM and IVC on Intracellular GSH and ROS Levels in IVP 4-Cell Embryos

The intracellular levels of GSH and ROS levels were analyzed in 4-cell IVP embryos. Oocytes were matured in IVM medium supplemented with or without melatonin, fertilized with thawed sperm, and cultured in IVC medium with melatonin for 48 h (IVM + IVC+, and IVC+ groups). Oocytes that were matured, fertilized, and cultured in the absence of melatonin constituted the controls. Ninety embryos at the 4-cell stage were evaluated in three replicates.

#### 2.11.4. Experiment 4. Effects of Melatonin during IVM and IVC on the DNA Damage of IVP Embryos

DNA damage was assessed in day 7 blastocysts produced in vitro. Oocytes that were matured in the presence or absence of melatonin were subjected to IVF with thawed sperm and cultured in an IVC medium containing melatonin (IVM + IVC+, and IVC+ groups). Controls were oocytes incubated without melatonin. Sixty blastocysts were analyzed in three replicates.

#### 2.11.5. Experiment 5. Effects of Melatonin during IVM and IVC on the Timing of In Vitro Development of IVP Embryos

This experiment determined the effect of melatonin on the developmental dynamics of IVP embryos. Melatonin was added to both the IVM and IVC media, or only to IVC medium (IVM + IVC+, and IVC+ groups). Oocytes that were matured, fertilized, and cultured in the absence of melatonin constituted the control group. The embryos were morphologically evaluated at 24, 48, 144, and 168 h of culture to assess the timing of embryonic development. A total of 868 immature oocytes were used in four replicates.

#### 2.11.6. Experiment 6. Effect of Melatonin during IVC on the In Vitro Development of In Vivo-Derived 4-Cell Embryos

In this study, the effect of the addition of melatonin to the IVC medium on the developmental ability and dynamics of 4-cell embryos produced in vivo was evaluated. The resistance to damage from low temperatures of the resultant blastocysts was also assessed. A similar number of 4-cell embryos collected from superovulated, and inseminated sows were cultured in an IVC medium with (IVC+ group; *n* = 70) or without (control; *n* = 76) melatonin. Embryos were visualized every 24 h from day 2 to day 5 of culture, and the numbers of the different classes of blastocysts (from early to hatched blastocysts) were recorded. Embryos achieving the expanded blastocyst stage (*n* = 144) were vitrified and stored in LN_2_. The post-warming embryo viability was determined after 24 h of culture. The experiment was performed in four replicates.

#### 2.11.7. Experiment 7. Effects of Melatonin during IVC on the Developmental Competence of Microinjected In Vivo-Derived Zygotes

In this experiment, the effects of melatonin on the developmental ability of microinjected zygotes produced in vivo were evaluated. A similar number of zygotes from each donor, which were collected from superovulated and inseminated sows, was microinjected and cultured for six days in an IVC medium supplemented with [IZ(IVC+) group; *n* = 217] or without [IZ(IVC-) group; *n* = 214)] melatonin in a total of nine replicates. Non-injected zygotes cultured in an IVC medium without melatonin (controls; *n* = 65) were used to determine the developmental capacity of in vivo-derived zygotes cultured in vitro. At 24 h and 168 h of culture, the embryos were visualized to evaluate the cleavage rates and blastocyst formation, and total efficiency rates, respectively.

### 2.12. Statistical Analysis

Differences between groups were examined with the IBM SPSS 24.0 Statistics package (SPSS, Chicago, IL, USA). The continuous variables are presented as the means ± standard errors of the means (SEM). The mean percentage ± SEM of the binary variables was obtained by calculating the percentage in every well of each group and each of the replicates. The normality of the variables was examined using the Shapiro–Wilk test. Groups were compared using an unpaired Student’s *t*-test (Experiments 1 and 6), ANOVA followed by the Bonferroni post-hoc test (Experiments 1, 2, 3, 4, 5, and 7), or a Fisher’s exact test (Experiment 6), as applicable. A *p* value < 0.05 was considered significant.

## 3. Results

### 3.1. Experiment 1. The Addition of Melatonin to IVC Medium, but Not to the IVM and/or IVF Media, Increases the Cleavage Rates and Blastocyst Efficiency of Inseminated Oocytes

**Figure 1:**
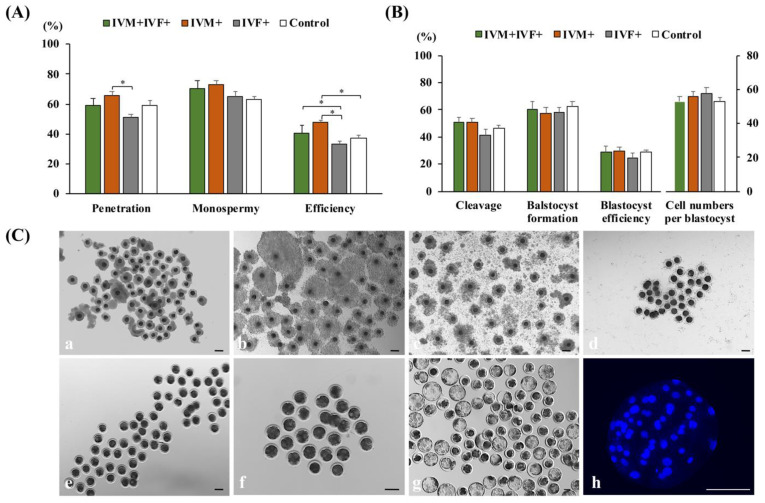
Effects of melatonin (1 nM) supplementation of in vitro maturation (IVM) and/or in vitro fertilization (IVF) medium on the development of porcine oocytes. (**A**) Fertilization parameters. IVM + IVC+: melatonin added to both IVM and IVF media (*n* = 132); IVM+: melatonin only added to IVM medium (*n* = 131); IVF+: melatonin only added to the IVF medium (*n* = 135); control: untreated (*n* = 134). Penetration: number of penetrated oocytes relative to the number of mature oocytes inseminated; monospermy: number of monospermic oocytes relative to the number of mature oocytes penetrated; efficiency: number of monospermic oocytes relative to the number of mature oocytes inseminated. Data are presented as the means ± SEM of six replicates. * *p* < 0.025. (**B**) Embryonic development parameters. IVM + IVC+ (*n* = 249); IVM+ (*n* = 247); IVF+ (*n* = 250); control (*n* = 258). The number of blastocysts evaluated to determine the total cell number in each group was 70, 76, 60, and 73, respectively. Cleavage: percentage of oocytes inseminated that developed to the 2- to 4-cell embryos at 48 h of culture; Blastocyst formation: percentage of cleaved embryos that progressed to blastocysts at day 7 of culture; blastocyst efficiency: percentage of oocytes inseminated that progressed to blastocyst. Data are presented as the means ± SEM of six replicates. (**C**) Representative images of immature oocytes (**a**), oocytes at 22 h and 44 h of maturation ((**b**,**c**) respectively), oocytes during the coincubation period (**d**), presumed zygotes after IVF (**e**), cleaved embryos, and blastocyst development at days 2 and 7 of culture ((**f**,**g**) respectively) and Hoechst-stained nuclei of an in vitro-produced blastocyst (**h**). Scale bar: 100 µm.

**Figure 2:**
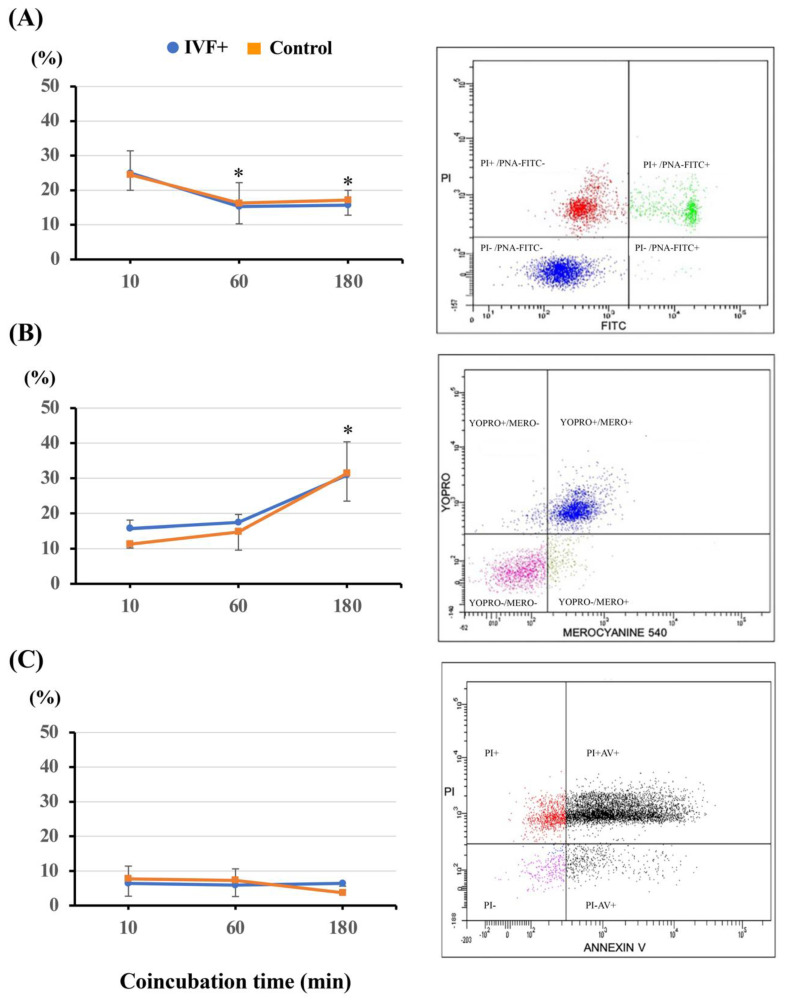
Effects of 1 nM melatonin addition to gamete coincubation medium (IVF+) on sperm parameters. (**A**) Sperm viability: viable sperm population exhibiting intact plasma and acrosomal membranes (PI−/PNA-FITC−); (**B**) Plasma membrane destabilization: viable sperm population with high fluidity of the plasma membrane (YOPRO−/MERO+); (**C**) Apoptosis: viable spermatozoa displaying early apoptotic-like changes (PI−/AV+). Gametes coincubated in the absence of melatonin constituted the control group. * Different from the first value (*p* < 0.05). Data are presented as the means ± SEM of three replicates.

**Figure 3:**
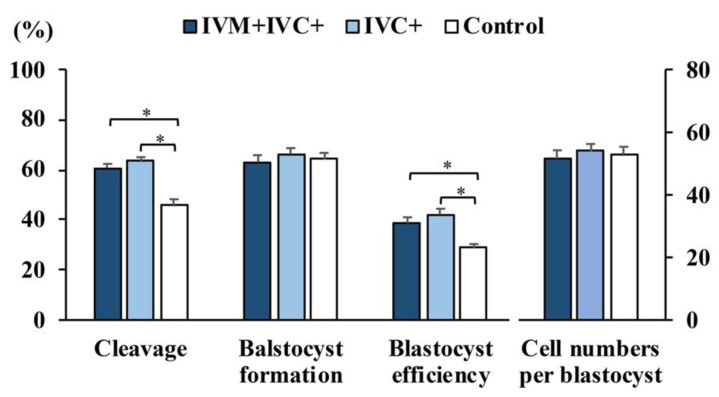
Effects of melatonin (1 nM) supplementation of in vitro maturation (IVM) and/or in vitro embryo culture (IVC) medium on the development of porcine zygotes. IVM + IVC+: melatonin added to both IVM and IVC media (*n* = 410); IVC+: melatonin added only to the IVC medium (*n* = 406); control: untreated (*n* = 409). The number of blastocysts evaluated to determine the total cell number in each group was 97, 105, and 93, respectively. Cleavage: percentage of oocytes inseminated that developed to the 2- to 4-cell embryos at 48 h of culture; blastocyst formation: percentage of cleaved embryos that progressed to blastocyst at day 7 of culture; blastocyst efficiency: percentage of oocytes inseminated that progressed to blastocysts. * *p* < 0.006. Data are presented as the means ± SEM of six replicates.

The rates of nuclear maturation in the oocytes matured in the presence of melatonin were comparable to those obtained in the control oocytes (*n* = 295; 91.8 ± 3.2% and 93.0 ± 1.7%, respectively). When only the IVM medium was supplemented with melatonin, the rate of total fertilization efficiency was higher (*p* < 0.001) than that of the controls (oocytes matured and fertilized without melatonin), but this effect disappeared when the oocytes were matured and fertilized in media containing melatonin (Figure 1A). Supplementation of melatonin in only the IVF medium did not alter either penetration and monospermic rates or fertilization efficiency compared with those of the control oocytes. Additionally, the embryonic development outcomes and the TCNs per blastocyst were comparable between the melatonin-exposed groups and the control group (Figure 1B). Melatonin did not affect sperm variables, including sperm viability, plasma membrane fluidity, and apoptosis, regardless of the coincubation period tested (Figure 2A–C). In contrast, although the TCN per blastocyst was not affected, the supplementation of IVC medium with melatonin increased (*p* < 0.001) both the cleavage rates and blastocyst efficiency rates compared to the controls, regardless of whether the IVM medium contained melatonin (Figure 3).

### 3.2. Experiment 2. Melatonin Supplementation during the IVC of Embryos Improved the Quality of the Resulting Blastocysts

**Figure 4:**
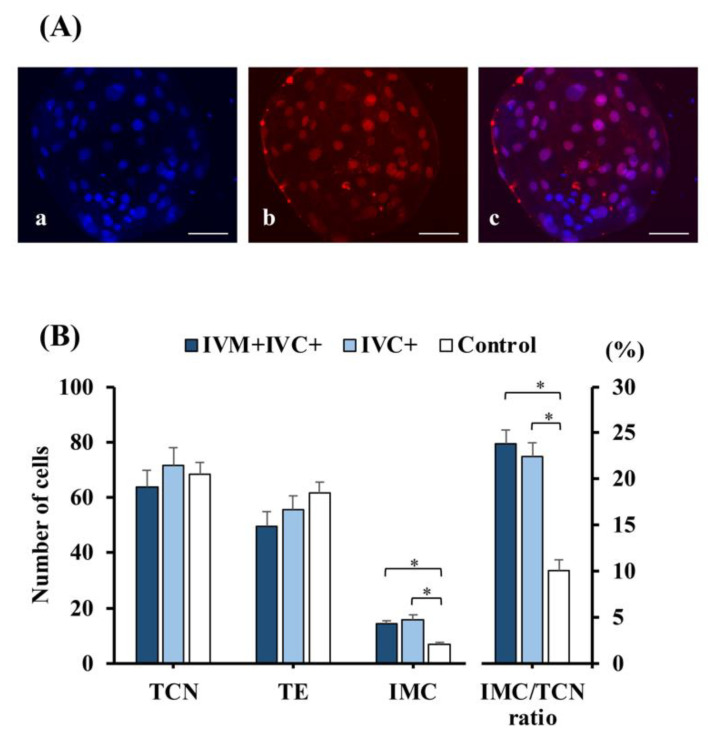
Differential staining of day 7 blastocysts produced in vitro. (**A**) Fluorescence microscopy image showing a blastocyst subjected to differential staining to determine the total cell number (TCN) and the number of trophectoderm (TE) cells, which were stained with Hoechst 33,342 (blue nuclei); (**a**) and an anti-CDX2 antibody (red nuclei); (**b**) Merged images (**c**) show the inner cell mass (ICM) and TE cells with blue and pink-red fluorescence, respectively. Scale bar: 50 µm. (**B**) Distribution of the different types of cells in blastocysts produced by adding melatonin (1 nM) to both the maturation and culture media (IVM + IVC+ group; *n* = 14), or only to the culture medium (IVC+ group; *n* = 15). Blastocysts derived from oocytes that were matured, fertilized, and cultured in media without melatonin supplementation were used as controls (*n* = 15). * *p* < 0.001. Data are presented as the means ± SEM (three replicates).

Melatonin supplementation of both IVM and IVC media or only IVC medium affected the quality of day 7 produced blastocysts. The TCN, the numbers of TE and ICM cells, and the ICM ratio per blastocyst are shown in Figure 4A,B. Although no differences in the TCN and the number of TE cells were observed among groups, the number of ICM cells and the ICM/TCN ratio were increased by more than two-fold in the groups exposed to melatonin compared to the control group (*p* < 0.001).

### 3.3. Experiment 3. Melatonin Supplementation during the IVC of Embryos Altered the Intracellular Levels of GSH and ROS Levels in IVP 4-Cell Embryos

**Figure 5:**
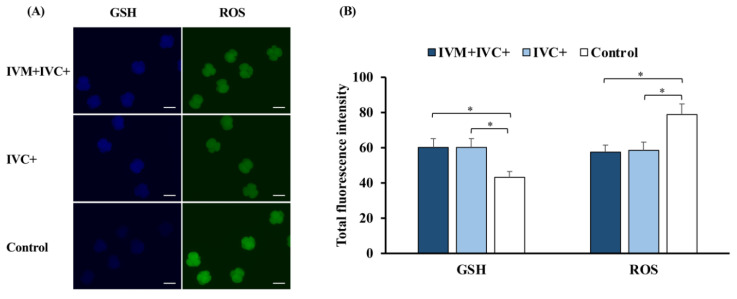
Effects of melatonin on intracellular glutathione (GSH) and reactive oxygen species (ROS) levels in in vitro-produced 4-cell embryos at 48 h of culture. (**A**) Representative images of embryos stained with CellTracker Blue (GSH) or with 2′,7′-dichlorodihydrofluorescein diacetate (ROS). Scale bar: 100 µm. (**B**) Effects of melatonin on GSH and ROS levels in embryos. Melatonin (1 nM) was added to both the maturation and culture media (IVM + IVC+ group; *n* = 28) or only to the culture medium (IVC+ group; *n* = 32). Oocytes that were matured, fertilized, and cultured in media without melatonin supplementation were used as controls (*n* = 30). * *p* < 0.03. Data are presented as the means ± SEM (three replicates).

The addition of melatonin to the IVC medium increased the GSH levels by ~40% (*p* < 0.03) and decreased the ROS levels by ~26% (*p* < 0.01) in the 4-cell IVP embryos compared to those in the control embryos, irrespective of whether the IVM medium contained melatonin (Figure 5A,B).

### 3.4. Experiment 4. Melatonin Supplementation during the IVC of Embryos Decreased the DNA Damage Rate in IVP Blastocysts

**Figure 6:**
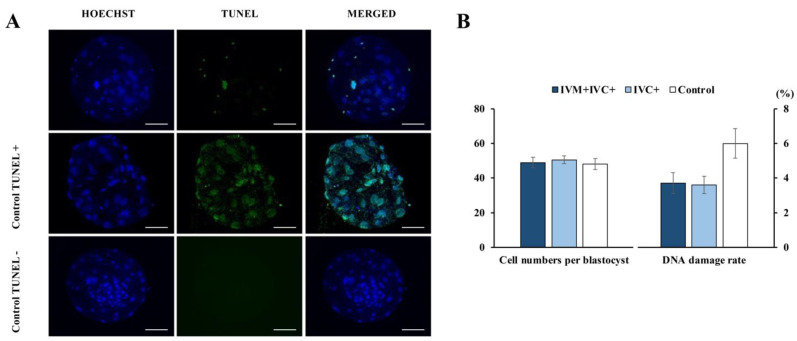
Detection of DNA damage in in vitro-produced Day 7 blastocysts. (**A**) Representative fluorescence images of the TUNEL assay. Hoechst (blue) and TUNEL (green) staining indicate the total cells and the cells with DNA damage, respectively. Scale bar: 50 µm. (**B**) Effects of melatonin on DNA damage rates in blastocysts. Melatonin (1 nM) was added to both the maturation and culture media (IVM + IVC+ group; *n* = 31) or only to the culture medium (IVC+ group; *n* = 30). Oocytes matured, fertilized, and cultured in media without melatonin supplementation were used as controls (*n* = 26). Data are expressed as the mean ± SEM (three replicates).

The DNA damage rate of blastocysts cultured in medium supplemented with melatonin decreased by ~40% compared with the blastocyst controls, regardless of whether the oocytes were matured with melatonin before IVF (Figure 6A,B).

### 3.5. Experiment 5. Melatonin Supplementation during the IVC Advanced the Development of IVP Embryos

**Figure 7:**
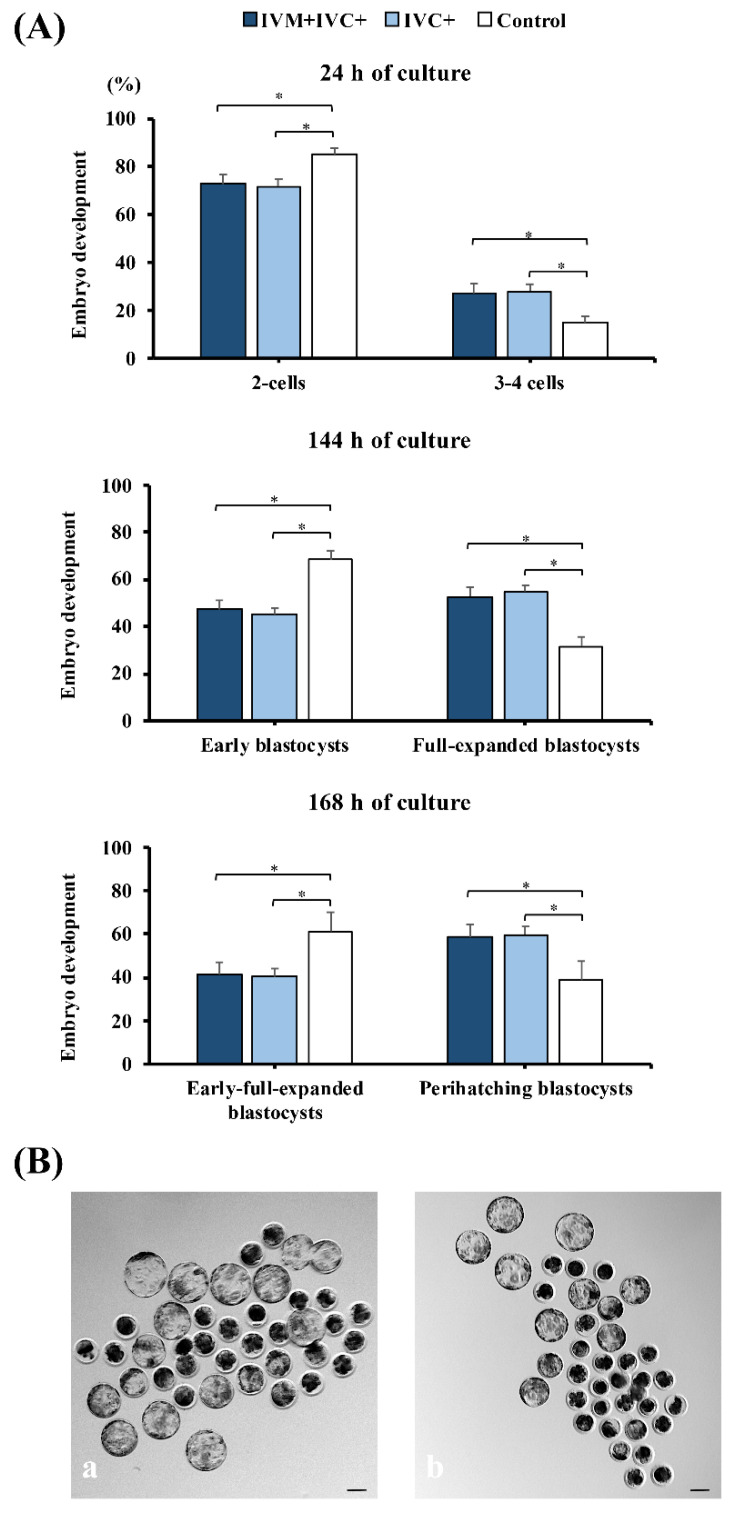
Embryonic developmental stages at 24 h, 144 h, and 168 h of culture were achieved by oocytes and embryos exposed or not exposed (control) to 1 nM melatonin. (**A**) Percentages of 3, 4-cell embryos at 24 h of culture from the total number of cleaved embryos at 48 h of culture and distribution of the different blastocyst stages reached in each group at 144 h and 168 h of culture, respectively. IVM + IVC+ group (*n* = 323): maturation and embryo culture performed in media supplemented with melatonin; IVC+ group (*n* = 310): embryo culture performed in medium containing melatonin. Control group (*n* = 295): maturation and embryo culture performed without melatonin. Perihatching blastocysts include hatching and hatched blastocysts. * *p* < 0.05. Data are presented as the means ± SEM (four replicates). (**B**) Representative images of blastocyst development at 168 h of culture in the presence (**a**) or absence (**b**) of melatonin. Scale bar: 100 µm.

The developmental dynamics of IVP embryos exposed to melatonin during IVM and IVC (IVM + IVC+ group) or only during IVC (IVC+ group) are presented in Figure 7. More than 25% of the cleaved embryos at 48 h post-insemination were already in the 3–4-cell stage at 24 h of culture in the groups supplemented with melatonin (27.2 ± 4.0% and 28.1 ± 2.9% for the IVM + ICV+ and IVC+ groups, respectively). These percentages were higher (*p* < 0.001) than those in control embryos (14.9 ± 2.7%). At 144 h of culture, supplementation with melatonin (IVM + IVC+ and IVC+ groups) resulted in >20 percentage points fewer (*p* < 0.002) early blastocysts (47.2 ± 3.9% and 45.1 ± 2.6%, respectively) and >20 percentage points more (*p* < 0.002) full and expanded blastocysts (52.8 ± 3.9% and 54.9 ± 2.6%, respectively) than those in the control group (68.3 ± 3.9% and 31.7 ± 3.9%, respectively). These differences remained at 168 h of culture, with the percentages of peri-hatching (hatching-hatched) blastocysts in the IVM + IVC+ (58.7 ± 5.7%) and IVC+ (59.7 ± 3.8%) groups >20 percentage points higher (*p* < 0.05) than those in the control group (38.7 ± 8.8%).

### 3.6. Experiment 6. Melatonin Supplementation of IVC Medium Accelerated the Dynamics of Embryonic Development but Did Not Alter the Developmental Capacity of In Vivo-Derived 4-Cell Embryos or the Resistance to Damage from Low Temperatures of the Resulting Blastocysts

**Figure 8:**
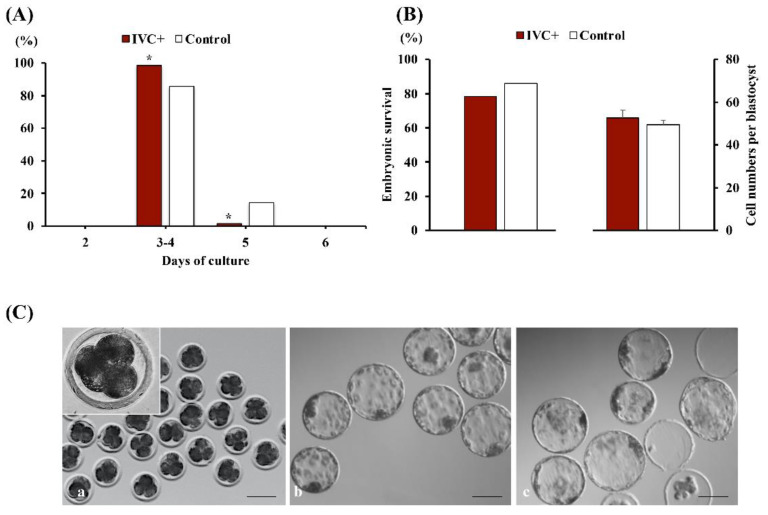
Effect of melatonin on the in vitro development of in vivo-derived 4-cell embryos. (**A**) Effects of the addition of 1 nM melatonin to the culture medium on the percentages of in vivo-derived 4-cell embryos developing to the expanded blastocyst stage or later stages at different days of culture. The embryos were cultured in culture medium with (IVC+; *n* = 70) or without (control; *n* = 76) melatonin. * *p* < 0.004 compared to the control group. (**B**) Post-warming survival and total cell number per blastocyst of vitrified-warmed expanded blastocysts formed from in vivo-derived 4-cell embryos cultured in a medium supplemented with (IVC+; *n* = 68) or without (control; *n* = 76) melatonin. Data are presented as percentages and means ± SEM (four replicates). (**C**) Representative images of in vivo-derived 4-cell embryos (**a**), fresh (**b**), and vitrified-warmed (**c**) blastocysts produced from the in vitro culture of in vivo-derived 4-cell embryos. Scale bar: 100 µm.

Treatment with melatonin did not alter the progression of in vivo-derived 4-cell embryos to the blastocyst stage, with blastocyst formation rates greater than 97% observed in both groups (IVC+ and control). However, the culture of embryos in a medium supplemented with melatonin resulted in 13 percentage points more embryos developing to at least the expanded blastocyst stage at days 3–4 of culture (98.5% and 85.5% for the IVC+ and control groups, respectively; *p* < 0.004) (Figure 8A). Post-warming survival rates and the TCN per warmed blastocyst was not altered by melatonin treatment (Figure 8B).

### 3.7. Experiment 7. The Addition of Melatonin to IVC Medium Did Not Improve the Developmental Capacity of Microinjected Zygotes Produced In Vivo

**Figure 9:**
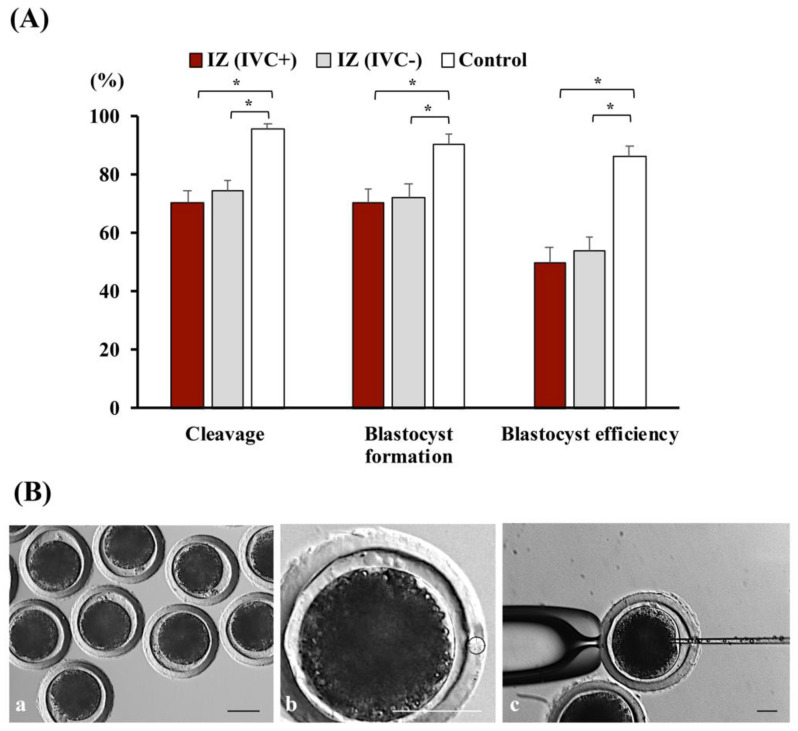
Effect of melatonin on the in vitro development of microinjected zygotes produced in vivo. (**A**) Cleavage, blastocyst formation, and blastocyst efficiency rates of in vivo-derived, injected zygotes (IZ) cultured for 168 h in a culture medium containing (IVC+; *n* = 217) or lacking (IVC−; *n* = 214) 1 nM melatonin. Noninjected in vivo-derived zygotes cultured in the absence of melatonin were used as controls (*n* = 65). * *p* < 0.001. Data are presented as the means ± SEM of six replicates. (**B**) Representative images of in vivo-derived zygotes (**a**) and microinjection procedure (**b**,**c**). Scale bar: 50 µm.

The microinjection procedure reduced (*p* < 0.001) cleavage, blastocyst formation, and blastocyst efficiency rates, whereas melatonin supplementation of IVC medium did not alter the developmental capacity of microinjected zygotes (Figure 9).

## 4. Discussion

To the best of our knowledge, this study is the first to evaluate the combined effect of melatonin supplementation of IVP media using oocytes fertilized in vitro in pigs. Optimal supplementation of IVM and/or IVF media with melatonin did not enhance embryonic development. However, when added to the IVC medium, melatonin not only improved porcine IVP outcomes but also accelerated the timing of embryonic development. In addition, we showed, for the first time, that melatonin added to the IVC medium failed to increase the developmental capacity of in vivo-derived 4-cell embryos and in vivo-derived CRISPR/Cas9 microinjected zygotes.

### 4.1. Melatonin Concentration

In the present study, we used 1 nM melatonin because that concentration was reported to be the optimum concentration in the maturation [22,27,52] and embryo culture [22,23,29,30] media for porcine embryos obtained via IVF, parthenogenesis, and SCNT. A melatonin concentration of 1 nM has also been found to be effective for bovine embryos fertilized in vitro [53,54,55,56,57,58,59,60], and for the IVC of in vivo-derived mouse embryos [61]. Furthermore, the concentration of melatonin was effective at protecting porcine oocytes from heat stress [62] and improving human oocyte tolerance to cold temperatures [63]. Therefore, our initial hypothesis was that 1 nM melatonin should appropriately maintain homeostatic ROS levels, preventing damage to gametes and embryos, which should increase the developmental capacity of porcine oocytes and embryos.

### 4.2. Melatonin in the IVM Medium and IVP of Embryos

The present study shows that the addition of melatonin to the IVM medium did not alter nuclear maturation, monospermic penetration, or subsequent embryonic development, disagreeing with some previous reports. Several factors exist that might explain this apparent discrepancy. First, the method of embryo production may affect the effectiveness of melatonin treatment during IVM. We used oocytes matured and fertilized in vitro, and our results are consistent with the only previous experiment using melatonin in the IVM medium and in vitro fertilized pig oocytes as an embryo production method [31].

Most studies indicating a positive effect of melatonin during maturation used parthenogenetic porcine oocytes [21,22,24,27,28,52], which are more sensitive to oxidative stress than the IVF oocytes [64]. In other species, such as cattle, the accumulating evidence that the addition of melatonin to the maturation medium is beneficial for the developmental competence of oocytes fertilized in vitro, although not always demonstrated [65,66,67,68], has encouraged researchers to use this antioxidant during the maturation of bovine oocytes [54,55,56,57,58,59,69,70,71]. All these findings suggest that, in addition to the source of the embryos, melatonin added to the IVM medium may function differently between species due to differences in the incubation times, chemical composition, and supplementation of media [72]. Second, we also speculated that the lack of effect of melatonin during maturation observed in our study is due to the optimal maturation protocol used in our lab (>90% of MII oocytes at 44 h of maturation) and that, therefore, the culture conditions were appropriate for the requirements of oocytes.

### 4.3. Melatonin in the Coincubation Medium and IVP of Embryos

Excessive ROS generation during gamete coincubation exerts harmful effects on fertilization and embryonic development [73,74]. We expected, therefore, that antioxidant supplementation of the IVF medium would decrease oxidative damage of gametes and enhance IVP efficiency. However, the supplementation of IVF medium with melatonin, regardless of whether the IVM medium contained melatonin, failed to improve the developmental competence of oocytes and the quality of the resulting blastocysts and did not affect sperm functionality, which agrees with bovine studies [75]. Nevertheless, most studies establishing the optimal melatonin concentration did not include specific dosages for the IVF step. Lower or higher concentrations of melatonin in the IVF medium than that used in our study might exert a favorable effect on fertilization and embryo production in the same way that a high melatonin concentration (1 mM) in bovine IVF medium significantly decreased blastocyst rates [75], possibly because that concentration of melatonin decreased ROS levels below the physiological levels necessary for normal fertilization [76].

### 4.4. Melatonin in the IVC Medium and IVP of Embryos

Our results clearly showed that the melatonin in the IVC medium enhances embryonic development, which is in line with previous reports using fertilized [29,30,77], parthenogenetic [22,78], or SCNT [23] oocytes. Melatonin in the IVC medium markedly augmented the number of ICM cells and the ICM/TCN ratio and, therefore, improved blastocyst quality, as the ICM number and the ICM/TCN ratio are common parameters indicative of embryo quality [79,80,81]. An appropriate ICM/TCN ratio (30% in in vivo-derived pig blastocysts [5,80]) is fundamental for the adequate establishment of gestation [82]. High ICM and low TCN in blastocysts are indicative of poor embryo quality and have been related to abnormalities in cloned animals [82]. The ICM/TCN ratio seems to be affected by culture environmental factors, such as oxygen concentration [80]. Although elevated concentrations of oxygen can be toxic to embryos due to the generation of ROS [83], in vitro culture of mammalian embryos is traditionally performed under atmospheric oxygen concentrations (~20%). In our study, like in previous reports [80], the ICM/TCN ratio was ~10% in in vitro-produced blastocysts cultured under classical conditions (NCSU23 medium and atmospheric oxygen concentrations of 20%). However, that ratio increased to approximately 25% in melatonin-treated blastocysts, indicating that the beneficial effect of melatonin on ICM number and ICM/TCN ratio might be explained by its antioxidant properties and protection of embryos from oxidative damage. Supporting this hypothesis, melatonin during IVC increased GSH levels and reduced ROS levels and DNA damage in embryos produced in vitro by IVF in our study. These findings are consistent with earlier studies using parthenogenic porcine embryos [20,26], IVF mouse embryos [84], and SCNT bovine embryos [81].

### 4.5. Melatonin in the IVC Medium and Kinetics of Embryonic Development

Fast-cleaving embryos present less apoptosis and higher blastocyst formation rates [85], TCNs, ICM/TCN rates, and fetal development [86]. Here, we described that melatonin supplementation of the IVC medium accelerated the timing of porcine embryonic development irrespective of whether melatonin was present in the IVM medium. This effect was evidenced by greater numbers of 3–4-cell embryos at 24 h of culture, full and expanded blastocysts at 144 h of culture, and perihatching blastocysts at 168 h of culture in melatonin-treated embryos compared to the controls. Moreover, melatonin accelerated the kinetics of development of in vivo-derived 4-cell embryos, with more embryos obtained at the expanded and hatching-hatched blastocyst stages after 3–4 days of culture in melatonin-treated than in nontreated embryos. These findings are consistent with a previous report in which melatonin positively influenced the kinetics of bovine blastocysts [87]. The kinetics of embryonic development are influenced by numerous factors of the IVC systems, including the incubation conditions and the composition of the culture media [88].

### 4.6. Melatonin in the IVC Medium and Development of In Vivo-Derived 4-Cell Embryos

In contrast to our results using in vitro-produced embryos, the presence of melatonin during IVC had no effect on the development of in vivo-derived 4-cell embryos. This finding was not surprising because an elevated percentage (>97%) of in vivo-derived 4-cell control embryos achieved the blastocyst stage, irrespective of melatonin treatment, which is consistent with previous results from our laboratory [6,89,90]. We should also consider that in vivo-derived embryos are exposed to endogenous melatonin during maturation, fertilization, and, at least, early embryonic development, as melatonin has been detected in follicular and oviduct fluids in pigs, rabbits, and humans [22,91,92]. Under these circumstances, additional melatonin supplementation during in vitro culture would not likely affect the development of these embryos.

We used the tolerance to cold temperatures as a criterion of blastocyst quality [87,93], and we did not observe differences in post-warming survival and TCN between melatonin-treated and untreated embryos during IVC. These results contrast those from previous studies in bovines showing that melatonin supplementation during IVC positively affected the tolerance to cold temperatures of in vitro-produced blastocyst [60,87]. In addition to the previous exposure of in vivo-derived embryos to endogenous melatonin mentioned above, this discrepancy could be explained by the notable differences between species in IVC protocols, which profoundly impact the embryo resistance to damage from low temperatures [94], and by the greater sensitivity to cold of IVP embryos compared with those derived in vivo [32,95]. Therefore, the effect of melatonin on embryo tolerance to cold temperatures may only be manifested in embryos of inferior quality, as is the case of IVP embryos.

### 4.7. Melatonin in the IVC Medium and the Development of In Vivo-Derived CRISPR/Cas9- Injected Zygotes

Melatonin in the culture medium also failed to improve the developmental competence of genome-edited in vivo-derived porcine zygotes. More than 50% of the CRISPR/Cas9-injected zygotes achieved the blastocyst stage after IVC, and this percentage was not increased by the addition of melatonin to the IVC medium. This study is the first to investigate the effects of melatonin on the in vitro development of edited zygotes. Previous studies have shown that melatonin during IVC supports the development of porcine and bovine SCNT embryos [20,23,81]. However, clear differences exist between SCNT embryos and edited zygotes. In SCNT, both ultraviolet irradiation during enucleation of oocytes and serum starvation of donor cells noticeably increase ROS production [96,97]. The effects of melatonin might be more evident in highly oxidizing environments when the damaging effects of ROS are intensified. Additionally, the higher quality and melatonin contents of the in vivo-derived zygotes used in our experiments compared with that of SCNT-reconstructed zygotes are potentially responsible for the lack of effect of melatonin on the development of zygotes to the blastocyst stage.

### 4.8. Future Research Directions

Summing up, the present study has provided strong scientific evidence and mechanistic insights into the pivotal role of melatonin as an extrinsic agent that not only decreases the intracellular levels of oxygen free radicals but also impedes/relieves the processes of oxidative stress in the in vitro cultured IVF-derived pig embryos. Considering the abovementioned findings, the advantageous effect of exogenous melatonin ought to be more widely adopted among laboratories working with IVP porcine (and other species) embryos, including those IVP embryos generated using intracytoplasmic sperm injection (ICSI) [98,99,100] and cloning by SCNT into enucleated oocytes [101,102,103].

## 5. Conclusions

Supplementation of melatonin in IVM medium, IVF medium, or both media did not increase fertilization or embryonic development parameters of IVP porcine oocytes. Melatonin also did not affect sperm functionality when added to the IVF medium. Melatonin supplementation of the IVC medium increased intracellular GSH levels, decreased intracellular ROS levels and DNA damage, accelerated embryonic developmental kinetics, and improved IVP outcomes and the quality, in terms of the number of ICM cells and the ICM/TCN ratio, of the blastocysts produced in vitro. Melatonin addition to the IVC medium also accelerated the timing of development of in vivo-derived 4-cell embryos but failed to improve their developmental potential or their quality in terms of tolerance to cold temperatures. Melatonin supplementation of the IVC medium also failed to increase the in vitro developmental capacity of genome-edited in vivo derived zygotes. It is therefore concluded that melatonin ought to be exogenously added to media for in vitro culture of IVM/IVF oocytes to enhance their development. In vivo-derived embryos do not benefit from such exogenous extra-exposure to the hormone.

## Data Availability

The data presented in this study are available in the article.

## Abbreviations

IVP	In vitro production
ROS	Reactive oxygen species
IVM	In vitro maturation
IVF	In vitro fertilization
IVC	In vitro culture
SCNT	Somatic cell nuclear transfer
ZGE	Zygote genome-editing
CRISPR/Cas9	Clustered Regularly Interspaced Short Palindromic Repeats/CRISPR-associated protein 9
COC	Cumulus-oocyte complex
TL-HEPES-PVA	Tyrode’s lactate-hydroxyethyl piperazineethanesulfonic acid- Polyvinyl alcohol
eCG	Equine chorionic gonadotropin
hCG	Human chorionic gonadotropin
BTS	Beltsville thawing solution
LEY	lactose egg yolk
BSA	Bovine serum albumin
PBS	Phosphate-buffered saline
ZP	Zona pellucida
NCSU-23	North Carolina State University-23 medium
M	Metaphase
MII	Metaphase II
PB	Polar body
TCN	Total cell number
PI	Propidium iodide
PNA-FITC	Fluorescein-conjugated peanut agglutinin
YOPRO	Yo-Pro-1
MERO	Merocyanine 540
AV	Annexin V
DMSO	Dimethyl sulfoxide
EG	Ethylene glycol
SOPS	Super open pulled straws
TE	Trophectoderm
ICM	Inner cell mass
GSH	Glutathione
TUNEL	Terminal deoxynucleotidyl transferase dUTP nick end labeling
LN_2_	Liquid nitrogen
SEM	Standard error of the mean
IZ	Injected zygotes
